# The Impact of Hypomania on Aerobic Capacity and Cardiopulmonary Functioning—A Case Report

**DOI:** 10.3389/fpsyt.2018.00729

**Published:** 2018-12-21

**Authors:** Aura Shoval, Hilary F. Armstrong, Julia Vakhrusheva, Jacob S. Ballon, Matthew N. Bartels, David Kimhy

**Affiliations:** ^1^Department of Rehabilitation and Regenerative Medicine, Columbia University, New York, NY, United States; ^2^Department of Psychiatry, Columbia University, New York, NY, United States; ^3^Department of Psychiatry and Behavioral Science, Stanford University, Stanford, CA, United States; ^4^Department of Rehabilitation Medicine, Albert Einstein College of Medicine, Bronx, NY, United States; ^5^Department of Psychiatry, Icahn School of Medicine at Mount Sinai, New York, NY, United States

**Keywords:** Schizophrenia, hypomania, aerobic fitness (VO_2_max), cardiopulmonary, mania and bipolar disorder, cardiopulmonar exercise testing, cardiopulmonary activity

## Abstract

**Background:** Hypomanic episodes are characterized by increased goal-directed behavior and psychomotor agitation. While the affective, cognitive, and behavioral manifestations of such episodes are well-documented, their physiological influence on aerobic capacity and cardiopulmonary functioning are unknown.

**Methods:** We describe a case report of an individual with schizophrenia who experienced a hypomanic episode while serving as a control participant (wait list) in a single-blind, randomized clinical trial examining the impact of aerobic exercise (AE) on neurocognition in people schizophrenia. As part of the trial, participants completed two scheduled clinical assessments and cardiopulmonary exercise tests (VO_2_max) at baseline and 12 weeks later at end of study. All participants received standard psychiatric care during the trial. Following a baseline assessment in which he displayed no evidence of mood lability, the subject returned on Week-12 for his scheduled follow-up assessment displaying symptoms of hypomania. He was able to complete the follow-up assessment, as well as third assessment 2 weeks later (Week-14) when his hypomanic symptoms ebbed.

**Results:** While not engaging in AE, the subject's aerobic capacity, as indexed by VO_2_max, increased by 33% from baseline to Week-12. In comparison, participants engaged in the aerobic exercise training increased their aerobic capacity on average by 18%. In contrast, participants in the control group displayed a small decline (−0.5%) in their VO_2_max scores. Moreover, the subject's aerobic capacity increased even further by Week-14 (49% increase from baseline), despite the ebbing of his hypomania symptoms at that time. These changes were accompanied by increases in markers of aerobic fitness including peak heart rate, respiratory exchange rate, peak minute ventilation, watts, and peak systolic blood pressure. Resting systolic and diastolic blood pressure, and peak diastolic blood pressure remained unchanged.

**Conclusions:** Our findings suggest that hypomania produce substantial increase in aerobic capacity and that such elevations may remain sustained following the ebbing of hypomanic symptoms. Such elevations may be attributed to increased mobility and goal-directed behavior associated with hypomania, as individuals in hypomanic states may ambulate more frequently, for longer duration, and/or at higher intensity. Our results provide a first and unique view into the impact of hypomania on aerobic capacity and cardiopulmonary functioning.

## Background

Hypomanic episodes are characterized by increased goal-directed behavior and psychomotor agitation manifested by more frequent pacing, fidgeting, and hand-wringing. While the cognitive, affective, and behavioral manifestations of such episodes have been documented extensively (1, 2), their influence on the physiological functioning of afflicted individuals is unknown. Specifically, the impact of hypomania on aerobic capacity and cardiopulmonary functioning is undetermined.

To address this issue, we describe a case report of an individual with schizophrenia, subject AA, who experienced a hypomanic episode while serving as a control participant in a single-blind, randomized clinical trial examining the impact of aerobic exercise (AE) on neurocognition in individuals with schizophrenia. As part of this study, participants were randomized to receive 12 weeks of regular psychiatric care (Treatment as Usual; TAU) or AE training in addition to TAU (3, 4). In this article, we report on subject AA's aerobic capacity and cardiopulmonary functioning before the trial (baseline), during the hypomanic episode (week 12), as well as 2 weeks later (week 14) when his hypomania symptoms ebbed. We characterized his performance and reviewed and contrasted it with the other study participants.

## Methods

The study was approved by the Columbia University's New York State Psychiatric Institute Institutional Review Board (NYSPI-IRB) and all participants signed a consent form. A written informed consent was obtained from the participant (subject AA) for the publication of this case report. All participants completed scheduled clinical, cognitive, and cardiopulmonary exercise tests (CPET) at baseline and follow-up (12 Weeks). Following approval by the NYSPI-IRB, subject AA completed a third CPET 2 weeks later (week 14).

### Measures

Detailed descriptions of the study rationale and procedures have been reported elsewhere (4–7). Briefly, aerobic capacity was determined by CPET to establish VO_2_max, considered the “gold standard” index of aerobic capacity. VO_2_max is an index of the maximum capacity of an individual's body to transport and use oxygen during incremental aerobic exercise, reflecting the individual's aerobic capacity level. All tests were completed on weekdays at ~10 a.m. and were performed on an electronically braked cycle ergometer (Ergometrics 800, SensorMedics Inc., Yorba Linda, CA) with a Viasys Encore metabolic cart (Viasys Corporation, Loma Linda, CA). Continuous 12-lead telemetry was monitored via CardioSoft electrocardiogram software (GE/CardioSoft, Houston, TX). Participants completed measurements of a 5-min resting baseline, 3-min of no-resistance warm-up, ramping exercise protocol of 10–15 watts to peak exercise with a target of exercise for 8–12 min. Exercise was terminated when the subject reached maximum capacity (VO_2_ plateau; 85% of maximal heart rate (HRmax; 220-age) (8); respiratory quotient ≥1.1; or self-reported exhaustion) (9). A 3-min active recovery period completed the test. We used VO_2_peak (ml/kg/min) scores in all analyses.

Additional cardiopulmonary variables were collected including heart rate (HR), respiratory exchange ratio (RER), resting and peak systolic and diastolic blood pressure (SBP, DBP), peak minute ventilation (VE), peak tidal volume (Vt), end tidal carbon dioxide pressure (PetCO_2_), rate of carbon dioxide production at peak (VCO_2_ L/min), and peak work rate (Watts). The clinical raters and technicians administering the CPET were blinded to the subjects intervention assignment and clinical status.

## Results

Subject AA is a slender 20-year-old, never married, male of Asian descent with a DSM-IV diagnosis of schizophrenia (onset at age 19). At the time of the study, he lived with relatives and had no family history of psychosis. At study entry his body mass index was 21.8 and he was prescribed Proloxin 37.5 mg injection every other week, lamotrigine 100 mg twice a day, hydroxyzine 50 mg twice a day, and benztropine 1 mg twice a day. He denied smoking. Subject AA endorsed moderately severe auditory hallucinations and religious delusions, but was clinically stable and reported to function well, just completing his freshman year in a local community college. Subject AA completed his baseline CPET, clinical, and cognitive assessments with no difficulties and was randomized to receive TAU. He displayed no mood lability during his baseline assessment. On week 12, upon arriving for the scheduled follow-up CPET, he displayed symptoms consistent with hypomania including elevated mood, flight of ideas, psychomotor agitation, and poor impulse control. Despite his condition, he completed his CPET, but given his clinical status, he was tested again 2 weeks later (Week 14), at which point his hypomanic symptoms were no longer present and he returned to his clinical baseline. His urine toxicology tests at baseline, Week 12, and Week 14 were all negative.

**Figure 1:**
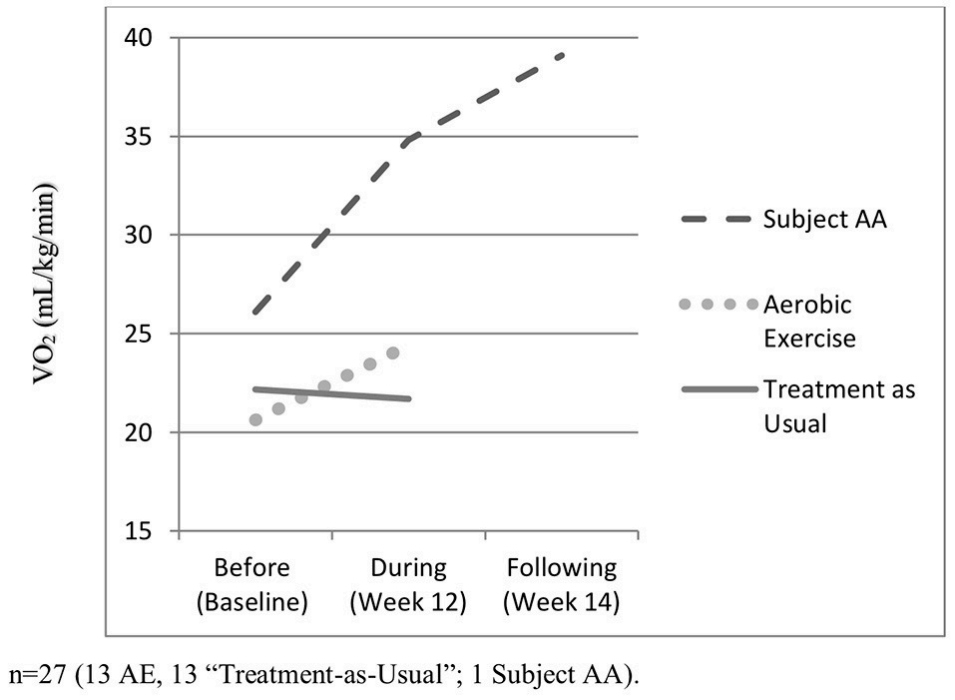
Aerobic capacity before, during, and post-hypomania.

Examination of changes in aerobic capacity from baseline to follow-up (Week 12) among all participants indicated the AE group increased their VO_2_max on average by 18%, compared to a small decline in the TAU group (−0.5%) (4). In comparison, Subject AA's VO_2_max increased by 33% during this period, although he denied engaging in any aerobic exercises in the preceding weeks. At Week 14, his aerobic capacity was elevated even further (49% increase from baseline), despite the ebbing of hypomania symptoms at that point (see Table 1 and Figure 1). His resting heart rate increased from 77 to 86 beats/min during hypomania and then went down to 70 beats/min following the hypomanic episode. A number of other cardiopulmonary variables increased from baseline to hypomania and post-hypomania periods including peak HR, respiratory exchange rate, peak minute ventilation, watts, and peak systolic blood pressure. PetCO2 decreased. These trends are all consistent with markers of increased aerobic fitness. In contrast, other parameters including resting systolic and diastolic blood pressure, as well as peak diastolic blood pressure remained unchanged.

**Table 1:** Subject AA cardiopulmonary functioning before, during, and post-hypomania.

	**Baseline (before hypomania)**	**Week 12 (during hypomania)**	**Week 14 (post-hypomania)**
VO_2_ Peak (ml/kg/min)	26.1	34.8	39.1
Resting heart rate (RHR)	77	86	70
Peak heart rate (PHR)	122	170	161
Respiratory exchange ratio (RER)	0.9	1.1	1.0
Peak minute ventilation (L/min)	42.6	65.8	73.4
Peak end tidal carbon dioxide pressure (PetCO_2_)	43.3	42.4	41.4
Peak wattage (watts)	100	154	155
Resting systolic blood pressure (RSBP)	115	118	121
Peak systolic blood pressure (PSBP)	157	198	183
Resting diastolic blood pressure (RDBP)	72	86	74
Peak diastolic blood pressure (PDBP)	75	78	80

To ensure that Subject AA's increase in VO_2_max was not related to higher workload, we examined his VO_2_ at the same workload (100 watts) to control for potential effort-related variability across sessions. His VO_2_ at 100 watts was lowest prior to the hypomanic episode (1.66 L/min), higher during (1.75 L/min), and highest following the episode (2.03 L/min). Examination of the performance at the same workload (100 watts) show that for Peak VO_2_, there was a larger component of volitional effect on conditioning. This view is corroborated by the similar trend in VO_2_max showing the lowest prior (26.1 kg L/min), higher during (34.8 kg L/min), and highest following the hypomanic episode (39.1 L/min). Likewise, VE also rose in a similar fashion and peak HR were elevated more at both follow up evaluations (Weeks 12 and 14).

## Discussion

To the best of our knowledge, the present report is the first characterization of the impact of hypomania on aerobic capacity and cardiopulmonary functioning. Our results suggest that episodes of hypomania produce substantial increases in aerobic capacity and that such elevations may be present even following the ebbing of hypomanic symptoms. Such increases may be attributed to heightened mobility and goal-directed behavior associated with hypomania, as individuals in hypomanic states may ambulate more frequently, for longer durations, and/or at higher intensity, resulting in increased VO_2_, peak wattage, as well as higher anaerobic threshold. Such episodes may also be associated with a greater ability to push harder on CPET, as indicated by higher workload (watts) with higher peak HR and VE in the latter tests. Thus, volitional aspects may also play a greater role in performance, necessitation careful review of CPET results of patients assessed during hypomanic states.

Overall, the improvement in subject AA's aerobic capacity, while not engaging in aerobic exercise, surpassed study participants engaging in a formal AE training program with a trainer (3 60-min sessions per week over 12 weeks). The improvement in other parameters such as workload and peak HR, support the view of hypomanic state-related increases in aerobic capacity, with the caveat that subject AA also gave a far higher effort with increased HR and minute ventilation. These indicators were even more elevated at Week 14. Overall, these findings are consistent with results from studies in the general population that indicate that increases in incidental physical activity (e.g., increased tapping of foot, pacing) correlate with higher VO_2_ max. For example, McGuire and Ross (10) have found that duration and intensity of incidental activities indexed by accelerometers were associated with higher VO_2_ max. Other authors have reported similar results, finding “fidgeting” and incidental physical activity increasing VO_2_max (11, 12).

VO_2_ is an important marker of physical health, as increased VO_2_ has been linked to early lower mortality (13, 14). Previous reports have documented significant lower aerobic fitness in individuals with schizophrenia (4) along with prevalence of early mortality among individuals diagnosed with schizophrenia, schizoaffective disorder, as well as bipolar disorder (15, 16). Thus, our findings of hypomanic episodes resulting in VO_2_max elevations provoke an intriguing question–does the experience of manic episodes and the resulting VO_2_ increases confer potential long-term protection with regard to early mortality risk? A recent meta-analysis has found no cardiorespiratory fitness differences between diagnostic subgroups of individuals with severe mental illness (e.g., schizophrenia, bipolar disorder, and major depressive disorder) (17). Previous reports have also documented significant elevations in early mortality among individuals with severe mental illness including those diagnosed with schizophrenia, schizoaffective disorder, and bipolar disorder (15, 16). Consistent with these findings, a review of medical records of 326 patients with a psychotic disorder treated at the Mayo Clinic in Minnesota between 1950 and 1980 found no significant difference in median survival for patients with schizophrenia vs. those with schizoaffective disorder (18). However, a population cohort study in Denmark (5,558,959 persons of which 261,887 persons had been admitted to a psychiatric hospital) found both females and males admitted with a diagnosis of schizophrenia had a higher mortality rate ratio of natural causes of death, compared to persons admitted with unipolar, bipolar, and schizoaffective disorders (19). Of note, natural causes of mortality comprised of cardiovascular diseases, respiratory diseases, endocrine and metabolic conditions, as well as old age and apoplexy, and malignant neoplasms. Thus, given the complexity of the longitudinal relationship between cardiopulmonary fitness and early mortality risk, the research literature at present does not provide conclusive support for the potential of manic episodes to confer protection for early mortality risk via temporal VO_2_ increases. Future studies should aim to elucidate this potential link, as it may inform the mechanisms associated with increased early mortality in individuals with severe mental illness.

The present report has a number of limitations. One limitation is the focus on a single individual. Secondly, the appraisal of (lack of) AE by Subject AA during the period prior to the follow-up assessments was based on his self-report, rather than via actigraph. Another limitation is the lack of follow-up assessments post the Week 14 assessment. Such information would have been valuable to characterize the timeline and long-term impact of hypomania on cardiopulmonary indicators. Finally, the changes in aerobic capacity and cardiopulmonary functioning may reflect, in part, Subject AA's young age, lower BMI, and relatively higher baseline aerobic capacity. Thus, the results should be interpreted with caution and future studies should aim to confirm our findings in larger and more diverse samples, including individuals who are older and have higher BMI. In contrast, the present report has a number of strengths including evaluation of naturally occurring hypomania that developed and resolved during “real world” functioning, a rigorous research assessment of clinical symptoms, as well as the use of CPET, a “gold standard” of aerobic capacity and cardiopulmonary functioning assessment.

In summary, the present report is the first characterization of changes in aerobic capacity and cardiopulmonary functioning associated with hypomanic episodes. Our results indicate that hypomanic episodes lead to substantial increases in aerobic capacity and improvements in cardiopulmonary functioning, and such increases may last for weeks and well-beyond the period of active hypomanic symptoms.

## Author Contributions

DK designed the study and wrote the protocol. MB and HA conducted the aerobic fitness and cardiopulmonary analyses, as well as provided the statistical data. JV conducted the diagnostic and clinical assessments. JB served as a medical director of the study and assisted with the monitoring of the participants' health. AS managed the literature searches, and along with DK, wrote the first draft of the manuscript. All authors contributed to and have approved the final version of the manuscript.

### Conflict of Interest Statement

The authors declare that the research was conducted in the absence of any commercial or financial relationships that could be construed as a potential conflict of interest.
